# Prediction of mortality in patients with secondary pulmonary embolism based on primary admission indication: A short communication

**DOI:** 10.17305/bb.2024.10481

**Published:** 2024-08-01

**Authors:** Martin J Ryll, Toby N Weingarten, Juraj Sprung

**Affiliations:** 1 Faculty of Medicine, Ludwig Maximilian University of Munich, Munich, Germany; 2 Department of Anesthesiology and Perioperative Medicine, Mayo Clinic College of Medicine and Science, Rochester, MN, USA

**Keywords:** Pulmonary embolism (PE), intensive care unit (ICU), critical care, mortality, Pulmonary Embolism Severity Index (PESI), simplified PESI (sPESI), modified sPESI (ICU-sPESI), Acute Physiology and Chronic Health Evaluation-IV (APACHE-IV), prediction of mortality

## Abstract

Secondary pulmonary embolism (PE) may significantly complicate the clinical course of intensive care unit (ICU) patients, creating the need for reliable stratification of severity and mortality risk in these patients. We evaluated the prediction of mortality in patients admitted to the ICU who subsequently developed a PE (i.e., secondary PE) using three PE-specific scores, the Pulmonary Embolism Severity Index (PESI), simplified PESI (sPESI), and modified sPESI (ICU-sPESI) and compared them to the gold standard for the assessment of ICU all-cause mortality, the Acute Physiology and Chronic Health Evaluation-IV (APACHE-IV). All critical care admission indications were grouped into four major categories: post-operative, cardiovascular, infectious (sepsis), and other. The APACHE-IV displayed better discriminative ability to predict in-hospital mortality than the PESI and ICU-sPESI, but these two scores still performed fair for the ICU admissions related to postoperative, cardiovascular, and other admission types. Meanwhile, the sPESI displayed poor predictive performance across all four admission categories. Notably, discriminatory performance for patients with an infection-related admission was consistently low regardless of which score was used.

## Introduction

The ability to quantify the risk of in-hospital mortality for patients with critical illness may result in cost savings and improvement of resource allocation [1]. The clinical presentation of patients with pulmonary embolism (PE) varies, from asymptomatic to complete cardiovascular collapse [2], and a large proportion of patients with PE require admission to the intensive care unit (ICU). Several PE-specific scores have been developed for predicting mortality in patients who present to the hospital with PE (i.e., primary PE), namely, the Pulmonary Embolism Severity Index (PESI) [3], simplified PESI (sPESI) [4], and ICU-modified sPESI (ICU-sPESI), specifically designed to improve prediction of mortality in critically ill patients [5, 6]. Details of the individual score components were provided in our earlier communication [5]. In the recent Biomolecules and Biomedicine report [6], we evaluated the predictive performance of these PE-specific tests for critically ill patients who were admitted to the ICU for an indication other than PE, but subsequently developed PE (i.e., secondary PE), and demonstrated that these tests had reduced accuracy compared to their predictions for primary PE [5, 6]. Since secondary PE in our previously reported cohort [6] occurred for a wide range of primary ICU admission diagnoses, we hereby explore whether the predictive performance differs depending on admission indications. The performance of PE-specific mortality risk scores was compared to the Acute Physiology and Chronic Health Evaluation-IV (APACHE-IV) score, which represents a gold standard for the prediction of all-cause mortality in ICU patients [7, 8].

## Materials and methods

For the full details on data source, cohort selection, data extraction, and processing, we refer the reader to our comprehensive description in our prior publication [6]. Briefly, using the eICU Collaborative Research Database (eICU-CRD) for 2014 and 2015 [9, 10], we identified 812 patients admitted to ICU with various indications (admissions unrelated to PE) and who were subsequently diagnosed with a secondary PE within 48 h after admission. These patients were classified into four broad categories according to the admission indications: a) post-operative, b) cardiovascular (non-surgical), c) infectious (e.g., most frequently sepsis), and d) any other indications (Table 1). The predictive accuracy of the APACHE-IV and three PE-specific risk-scores (PESI, sPESI, and ICU-sPESI) were compared using the area under the receiver operating characteristic curve (AUROC) [11]. AUROCs were descriptively interpreted as follows: AUROC ≥0.9 was considered excellent, ≥0.8 to <0.9 good, ≥0.7 to <0.8 fair, ≥0.6 to <0.7 poor, and <0.6 non-discriminatory [12, 13]. Statistical analyses were performed with Python v.3.9 (Python Software Foundation, Wilmington, Delaware, USA).

**Table 1:** Primary admission indications and in-hospital mortality rate among the four major admission indications. Critically ill patients with a secondary PE were grouped into four major admission indications, each exemplified by the most common diagnoses

**Admission indications**	**In-hospital mortality [95% CI]**
Post-operative (*n* ═ 65)	18.5% [9.0%–27.9%]
Gastrointestinal	
Neurosurgical	
Cardiovascular	
Thoracic	
General/endocrine/otorhinolaryngological	
Infectious (*n* ═ 260)	18.5% [13.7%–23.2%]
Sepsis, pulmonary/pneumonia	
Sepsis, renal/urinary tract infections	
Sepsis, gastrointestinal	
Sepsis, cutaneous/soft tissue	
Sepsis, other/unknown	
Endocarditis	
Encephalitis/meningitis	
Cholangitis	
Cardiovascular (*n* ═ 214)	21.5% [16.0%–27.0%]
Cardiac arrest/MI	
Dysrhythmia (supraventricular, ventricular, etc.)	
Congestive heart failure	
Unstable angina	
Aortic dissection	
Other (*n* ═ 273)	16.1% [11.8%–20.5%]
Emphysema/bronchitis	
Respiratory arrest	
ARDS/pleural effusions	
Cerebrovascular accident/stroke	
Gastrointestinal bleeding	
Acute renal failure	
Intracranial hemorrhage	
Cancer/neoplasm	

ARDS: Acute respiratory distress syndrome; MI: Myocardial infarction; UTI: Urinary tract infection; PE: Pulmonary embolism.

## Results

**Figure 1.**
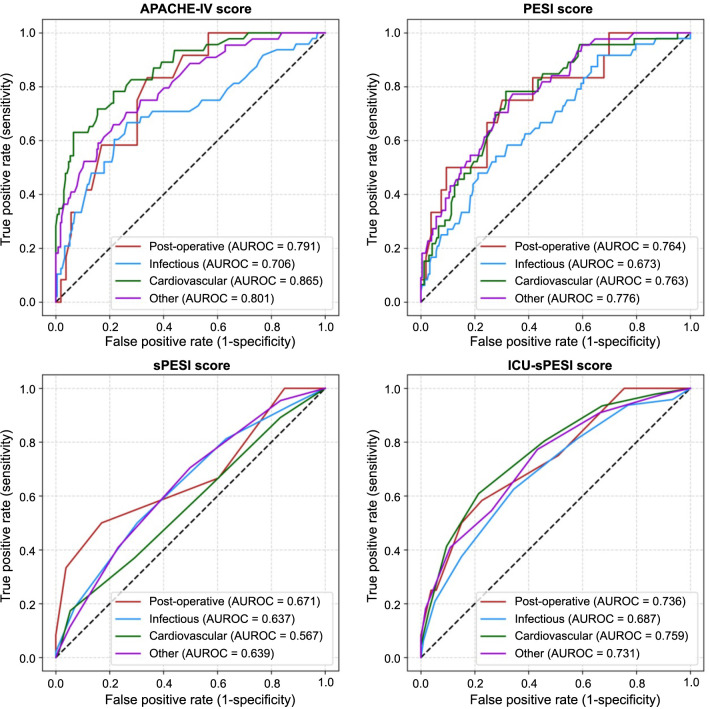
**Receiver operating characteristic curves for the different subgroups of primary ICU admission diagnoses.** APACHE-IV: Acute Physiology and Chronic Health Evaluation-IV; AUROC: Area under the receiver operating curve; ICU: Intensive care unit; PESI: Pulmonary Embolism Severity Index score; sPESI: Simplified PESI; ICU-sPESI: ICU-modified sPESI.

Table 1 provides an overview of the four categories of admission indications and their overall in-hospital mortality rates, as well as examples of individual admission diagnoses. Overall, mortality was similar in all categories, with intersecting confidence intervals, highest for cardiovascular (21.5%, 95% CI [16.0%–27.0%]), and lowest for other admissions (16.1%, 95% CI [11.8%–20.5%]). Median [IQR] APACHE-IV, PESI, sPESI, and ICU-sPESI scores for the admission categories are shown in Table 2. Compared to survivors, non-survivors had higher risk scores regardless of admission category. AUROC analyses according to admission indication are detailed in Figure 1 and Table 3. In particular, the sPESI performed poorly for postoperative, infectious, and “other” admissions and had a non-discriminatory performance for cardiovascular admissions. In addition, cardiovascular admissions displayed the largest difference between the APACHE-IV and the PE-specific risk scores, with better performance noted for APACHE-IV compared to PE-specific scores (APACHE-IV vs PESI, *P* ═ 0.018; APACHE-IV vs sPESI, *P* < 0.001, APACHE-IV vs ICU-sPESI, *P* ═ 0.033). Despite the similar overall mortality, all scores, including the APACHE-IV, performed worse for infectious admissions (fair for APACHE-IV, AUROC ═ 0.706, and poor for PESI, sPESI and ICU-sPESI, AUROCs 0.673, 0.637, 0.687, respectively). Notably, by adding only three binary variables to the sPESI for calculating the ICU-sPESI, this modified score performed similarly well in comparison to the more complex PESI score across all admission indications.

**Table 2:** PESI, sPESI, and ICU-sPESI scores in the subgroups as classified by primary ICU admission indications

**Subgroups/scores**	**All patients (*N* ═ 812)**	**Survivors (*n* ═ 662)**	**Non-survivors (*n* ═ 150)**	***P* values**
*Post-operative (n ═ 65)*				
APACHE-IV	54.0 [36.0–77.0]	48.0 [32.0–66.0]	78.0 [57.8–91.2]	0.002
PESI	132.0 [99.0–178.0]	124.0 [83.0–161.0]	184.0 [152.5–216.2]	0.005
sPESI	2.0 [1.0–2.0]	2.0 [1.0–2.0]	2.5 [1.0–4.0]	0.056
ICU-sPESI	3.0 [2.0–4.0]	3.0 [2.0–3.0]	4.5 [2.8–5.5]	0.010
*Infectious (n ═ 260)*				
APACHE-IV	60.5 [46.0–80.0]	58.0 [45.0–75.0]	81.5 [55.8–98.5]	<0.001
PESI	143.5 [106.0–179.0]	140.5 [100.8–170.0]	170.0 [133.8–203.8]	<0.001
sPESI	2.0 [1.0–3.0]	2.0 [1.0–3.0]	2.5 [2.0–3.0]	0.002
ICU-sPESI	3.0 [2.0–4.0]	3.0 [2.0–4.0]	4.0 [3.0–5.0]	<0.001
*Cardiovascular (n ═ 214)*				
APACHE-IV	56.5 [42.0–82.0]	50.0 [38.8–65.0]	112.0 [71.5–146.0]	<0.001
PESI	130.0 [93.0–162.8]	118.5 [89.8–152.0]	162.5 [144.2–197.2]	<0.001
sPESI	2.0 [1.0–3.0]	2.0 [1.0–3.0]	2.0 [1.0–3.0]	0.151
ICU-sPESI	3.0 [1.0–4.0]	2.0 [1.0–3.0]	4.0 [3.0-5.0]	<0.001
*Other (n ═ 273)*				
APACHE-IV	51.0 [40.0–70.0]	49.0 [39.0–67.0]	81.5 [59.5–110.5]	<0.001
PESI	137.0 [92.0–172.0]	125.0 [85.0–163.0]	178.0 [147.8–211.5]	<0.001
sPESI	2.0 [1.0–3.0]	1.0 [1.0–2.0]	2.0 [1.0–3.0]	0.002
ICU-sPESI	2.0 [1.0–4.0]	2.0 [1.0–4.0]	4.0 [3.0–5.0]	<0.001

Data are represented as median [IQR]. APACHE-IV: Acute Physiology and Chronic Health Evaluation-IV; ICU: Intensive care unit; PESI: Pulmonary Embolism Severity Index score; sPESI: Simplified PESI; ICU-sPESI: ICU-modified sPESI.

**Table 3:** AUROC *P* value comparisons of the different scores in each subgroup of primary ICU admission diagnoses

**Indications for ICU admission**	**APACHE-IV vs PESI**	**APACHE-IV vs sPESI**	**APACHE-IV vs ICU-sPESI**	**PESI vs sPESI**	**PESI vs ICU-sPESI**	**ICU-sPESI vs sPESI**
Post-operative	0.767	0.297	0.602	0.288	0.700	0.293
Infectious	0.506	0.259	0.724	0.449	0.710	0.195
Cardiovascular	**0.018**	**<0.001**	**0.033**	**<0.001**	0.927	**<0.001**
Other	0.596	**0.004**	0.159	**0.002**	0.215	**0.010**

Bolded values are statistically significant. APACHE-IV: Acute Physiology and Chronic Health Evaluation-IV; AUROC: Area under the receiver operating curve; ICU: Intensive care unit; PESI: Pulmonary Embolism Severity Index score; sPESI: Simplified PESI; ICU-sPESI: ICU-modified sPESI.

## Discussion

We examined four mortality prediction scores and demonstrated their different discriminative ability to predict in-hospital mortality of secondary PE depending on the primary admission indication. In patients with secondary PE, APACHE-IV was a better prognosticating instrument than the three PE-specific prediction scores regardless of the nature of ICU admission. The discriminatory ability of the PESI and ICU-sPESI was still within the acceptable range for postoperative, cardiovascular, and other admissions, but was less accurate for infectious admissions. Better performance of APACHE-IV compared to the PE-specific scores is likely related to the APACHE-IV covering a wide range of differentially weighted clinical variables integrated into complex algorithms [8]. In contrast, the simpler PE-specific scales were designed to include signature features associated with primary PE and these relatively focused inclusion criteria are likely responsible for the observed reduction of predictive precision after secondary PE. It is important to note that when the ICU admission was related to infection, all four scores underperformed. This may be expected because sepsis represents a systemic, multi-etiological disorder with a wide variability of clinical presentations and unpredictable responses to treatment. Furthermore, mortality prediction scores are typically built by collecting clinical variables early and in a relatively short timeframe (e.g., APACHE-IV variables are collected within the first 24 hours of admission). In contrast, sepsis can change its clinical course rapidly, progressing from mild to severe over a short period of time, thus being improperly represented. All this renders the prediction of mortality from sepsis more difficult, even for a score as comprehensive as the APACHE-IV. Consistent with this finding, several previous studies demonstrated that the ability of general scoring systems to predict outcomes in septic patients is frequently unreliable compared to diseases that affect specific organ systems [14–16].

## Conclusion

In our study, APACHE-IV had the best ability to predict all-cause in-hospital mortality in critically ill patients with a secondary PE. However, PESI and ICU-sPESI still offered a fair predictive ability for postoperative, cardiovascular, and other admission indication categories. In contrast, sPESI displayed a poor performance throughout. Notably, discriminatory performance for patients with an infectious admission indication was low regardless of which score was used. More studies are needed to improve the accuracy of outcome prediction scores; however, as stated decades ago by Becker and Zimmerman [17], “even with the highest degree of precision, such predictions are only useful in support of, and not as a substitute for, good clinical judgment.”

## Data Availability

For access to the eICU-CRD dataset, please review https://eicu-crd.mit.edu/. The code for data extraction and data analysis can be found at https://github.com/RyllMartin/eICU_ICU_sPESI_validation_secondary_PE.
